# ASO Author Reflections: Shifting Age Demographics in Prostate Cancer Surgery: Insights from 17 Years of German Data

**DOI:** 10.1245/s10434-025-18827-z

**Published:** 2025-12-06

**Authors:** Martin Baunacke, Johannes Huber, Lennard Haak, Christian Thomas, Christer Groeben

**Affiliations:** 1 Department of Urology, University Hospital Carl Gustav Carus, TU Dresden, Dresden, Germany; 2 Department of Urology, Department of Urology, University of Heidelberg, Heidelberg, Germany; 3 Department of Urology, Philipps-University Marburg, Marburg, Hessen, Germany

## Past

The study addresses the clinical question of how increasing patient age affects the utilization and outcomes of radical prostatectomy (RP) for prostate cancer. As life expectancy continues to rise in Western countries, more elderly men seek curative treatment.^1^ However, most previous studies were limited to single institutions and smaller cohorts, providing little insight into nationwide trends.^2,3^ The main scientific problem was the lack of population-based data examining whether older patients (particularly those aged ≥ 75 years) increasingly undergo RP and how age impacts perioperative outcomes such as mortality, transfusion rates, and hospital stay length.

## Present

Using national hospital billing data from Germany (2006–2022; 444,102 cases), the study demonstrates a clear and continuous increase in the mean age of RP patients, with the proportion of men ≥ 75 years quadrupling from 3 to 12% over 17 years. Although older patients experienced higher in-hospital mortality (0.3% versus 0.1%), higher transfusion rates (5% versus 2%), and longer hospital stays (8.15 versus 7.57 days), these differences were statistically significant but clinically minor. Importantly, the study shows that RP, including robotic-assisted approaches, remains a feasible and safe option for well-selected elderly patients. It also highlights a shift in treatment decision-making—from rigid chronological age criteria to biological fitness and comorbidity assessment—reflecting increased flexibility in guideline adherence.^4^

## Future

Future research should focus on functional outcomes (continence and erectile function) in elderly patients, as these aspects substantially affect quality of life but remain underexplored in national data.^5^ Studies should also assess whether the growing number of elderly RP patients impacts overall postoperative recovery and healthcare resource allocation. Furthermore, given the rising demand for surgical treatment among older men alongside a declining medical workforce, there is a need for strategic planning to ensure that these patients receive optimal care in high-volume, experienced centers. Future investigations integrating geriatric assessment tools and long-term oncological outcomes will be crucial for refining patient selection and treatment guidelines.

## References

[1] Culp MB, et al. Recent global patterns in prostate cancer incidence and mortality rates. *Eur Urol*. 2020;77(1):38–52. 31493960 10.1016/j.eururo.2019.08.005

[2] Greco KA, et al. Robot-assisted radical prostatectomy in men aged > or = 70 years. *BJU Int*. 2009;104(10):1492–5. 19583731 10.1111/j.1464-410X.2009.08718.x

[3] Ubrig B, et al. Outcome of robotic radical prostatectomy in men over 74. *J Endourol*. 2018;32(2):106–10. 29232985 10.1089/end.2017.0512 PMC5813731

[4] Baunacke M, et al. Increasing age at radical prostatectomy: a total population analysis in Germany from 2006 to 2022. *Ann Surg Oncol*. 2025. 10.1245/s10434-025-18740-5. 41261268 10.1245/s10434-025-18740-5 PMC12901175

[5] Herkommer K, et al. Health-related quality of life after radical prostatectomy depends on patient’s age but not on comorbidities. *Urol Oncol*. 2015;33(6):266 e1-7. 25847625 10.1016/j.urolonc.2015.03.005

